# The Impact of Third-Party Information on Trust: Valence, Source, and Reliability

**DOI:** 10.1371/journal.pone.0149542

**Published:** 2016-02-16

**Authors:** Christiane Bozoyan, Sonja Vogt

**Affiliations:** 1 Institute of Sociology, Ludwig-Maximilians-University Munich, Munich, Germany; 2 Department of Economics, University of Zurich, Zurich, Switzerland; 3 Laboratory for Social and Neural Systems Research, University of Zurich, Zurich, Switzerland; Technical University Darmstadt, GERMANY

## Abstract

Economic exchange between strangers happens extremely frequently due to the growing number of internet transactions. In trust situations like online transactions, a trustor usually does not know whether she encounters a trustworthy trustee. However, the trustor might form beliefs about the trustee's trustworthiness by relying on third-party information. Different kinds of third-party information can vary dramatically in their importance to the trustor. We ran a factorial design to study how the different characteristics of third-party information affect the trustor’s decision to trust. We systematically varied unregulated third-party information regarding the source (friend or a stranger), the reliability (gossip or experiences), and the valence (positive or negative) of the information. The results show that negative information is more salient for withholding trust than positive information is for placing trust. If third-party information is positive, experience of a friend has the strongest effect on trusting followed by friend’s gossip. Positive information from a stranger does not matter to the trustor. With respect to negative information, the data show that even the slightest hint of an untrustworthy trustee leads to significantly less placed trust irrespective of the source or the reliability of the information.

## Introduction

Economic exchanges with strangers are becoming more and more frequent due to the growing number of internet transactions. Online markets have become very popular, and thousands of anonymous buyers and sellers are engaged in them on a daily basis. Paypal International alone generated 955 million USD in revenue in the first three months of 2014, i.e. 6688 USD in total payments every second. Internet transactions are mostly not face-to-face, often in different regions or countries, and usually without clear legal enforcement. Sellers typically are protected from opportunistic buyers by demanding advance payment. Thus they receive money first and then send out the product. However, online transactions bring uncertainty on the side of the buyer because she cannot know whether the product satisfies her expectations before paying for it. Sellers might not have incentives to invest in a good reputation because they do not share a common future with the buyer. Thus buyers bear the risk of exploitation [1]. Nevertheless, every day thousands of people place trust in an online seller.

Let us consider the example of Simon, a young man interested in photography with quite a selection of camera equipment. After some time, Simon decided that a couple of the lenses he had bought were not useful for the kind of photography he was interested in. Therefore, Simon wanted to sell the lenses on an informal online photo forum he occasionally visited. Within a couple of days, Simon managed to sell his lenses to strangers who transferred the money in advance before Simon shipped the lenses. Thus, the buyers did not inspect the quality of the lens before paying. Buyers did not know whether Simon was a trustworthy seller. Moreover, the forum did not provide buyer protection or an official rating system. Why did some people trust Simon, transfer the money in advance, and expect to receive a satisfying product, while others decided not to pay for such a delicate product without inspecting its quality first and consequently withheld trust?

Trust has always been considered an important ingredient of economic exchange as it facilitates transactions among both individuals and organizations in the absence of complete contracts [2, 3, 4, 5]. Coleman defines trust as an action that involves the voluntary placement of resources at the disposal of a trustee with no enforceable commitment from the trustee. Trust creates mutual benefits if the trustee honors trust and an individual loss to the trustor if the trustee dismisses trust and is opportunistic [6]. The internet transaction we described above resembles such a trust problem in that a buyer trusts if she voluntarily places resources at the disposal of the seller without any legal commitment from the latter. The act of trust is associated with an expectation by the trustor that the act will pay off in terms of the trustor’s goals [6, 7, 8]. In situations where the trustor cannot know whether she encountered a trustworthy trustee, she nevertheless forms beliefs about trustworthiness [9, 10]. Ermisch and colleagues describe the situation, ‘these beliefs are […] based, at least in part, on learning through experience. In most real life circumstances these beliefs refer to specific people or groups of people whom we believe share certain trust warranting properties’ [11].

Beliefs about others are crucial in social dilemmas even though social dilemmas have a dominant strategy [12]. Research has shown that many people cooperate conditional on a sufficiently strong belief that other people also cooperate [13, 14]. Therefore, one way to solve a trust problem is to improve the trustor’s information about the trustee. This helps the trustor update her beliefs regarding the trustee’s trustworthiness. Considering the example of Simon again, it could be that some buyers knew others who bought products from Simon before and were satisfied, while other buyers heard negative gossip about doing business with Simon in the online forum. Thus, the buyers had different kinds of information they used to update their beliefs regarding Simon’s trustworthiness. The current paper focuses on the question, what kind of information is important for a buyer and consequently affects her decision to trust the seller in an online transaction?

In big online communities, official reputations systems often provide information about the trustee to the trustor [15, 16, 17, 18, 19]. However, the internet provides plenty of information that is not regulated by an official agency, information that comes directly from other online users who can give their opinion regarding certain transactions. The huge transaction platform Craigslist, for instance, states on its homepage that Craigslist is not involved in any transaction, does not handle payments, provide escrow, buyer protection or seller certification (www.craigslist.org/about/scams). Nevertheless, Craiglist has more than 50 billion page views per month. Studying third-party information that is not regulated by an official reputation system has the advantage that the significance of the third-party information to the trustor is not mediated by a trustor’s general trust in the online reputation system or the associated company [20]. Third-party information, which is not regulated by an official agency, directly affects a trustor’s belief regarding the trustee’s trustworthiness. Often the risk in trusting a trustee with whom the trustor has no shared past is mitigated by information provided by a third party [21].

Earlier research has documented very well the effects of third-party information on trust in dyads. If the trustor is informed about the trustee’s past behavior, and if this is common knowledge between the trustor and the trustee, then trust as well as trustworthiness increase [22, 23, 24]. Most research varies the amounts of information regarding the seller’s history [25, 26]. Duffy et al. [27] and Keser [28] study whether the length of the reputation record matters for the trustor to place trust. Keser does not find an effect of the length of the reputation record on placing trust, assuming a longer record is more valuable to the trustor, while Duffy and colleagues do find such an effect.

In spite of the focus on the length of the reputation record, informal third-party information can vary dramatically in its effects on the trustor for other reasons [29]. Some information is more helpful than other information. Information might come from the trustor’s friend who has heard some gossip about the trustee. Information can also be based on the experiences of a stranger. Sometimes information regarding the trustee is positive and sometimes negative, i.e. based on negative or positive gossip or experiences from a friend or a stranger. It is not well understood how multidimensional third-party information affects the trustor’s beliefs regarding the trustee’s trustworthiness. Different characteristics of third-party information can have countervailing effects, and it is impossible to predict an order for all combinations of characteristics a priori. A systematic study is needed in order to understand how the different characteristics of third-party information combine to affect the trustor’s decision to trust. Accordingly, we developed a unique vignette study that systematically varies the characteristics of third-party information for the trustor in three critical ways hypothesized to affect trust.

Namely, in our study we manipulate the valence, source, and reliability of third-party information. It is well known that information gathered by one’s self has a bigger effect on trust decisions than information provided by a third party [30, 31]. However, the significance of third-party information also depends on the relationship between the trustor and the third party. We therefore manipulate the source of the information as either a stranger or a friend of the trustor [32]. Moreover, it matters whether third-party information is reliable. Thus, we study information based on either the direct experiences of the third party with the trustee or on mere gossip heard by the third party [33]. Finally, the valence of third-party information affects its importance to the trustor, and therefore we distinguish between positive and negative information [34, 35].

Outside the laboratory the nature of third-party information is difficult to study. A factorial design study is ideally suited for this because the method allows you to control the different dimensions independently. The current paper presents a novel factorial design that allows us to vary the salience third-party information has for the trustor in terms of the information’s valence, source, and reliability. We vary the different dimensions independently to study their main effects on trust. Moreover, the factorial design also allows us to examine any interactions among the different dimensions of third-party information.

## Theory

Third-party information can come from a friend or a stranger, can be positive or negative, and can be based on personal experiences or gossip. We are interested how variation in the salience of third-party information to the trustor affects the decision to trust. We discuss first the isolated effects of valence, source, and reliability and compare their effects to the baseline condition without third-party information. We then discuss potential interactions between the three dimensions in comparison to the baseline condition with no third-party information.

### Valence: positive versus negative information

The third party can give positive or negative information regarding the trustee. Blau [34] stressed that it takes time to develop a positive reputation, whereas it is easy to destroy one. The information that a trustee has dishonored trust is a clear signal that the trustee does not belong to the trustworthy trustees who honor trust in any occasion. The information that a trustee has so far honored trust is less clear because untrustworthy trustees also have an incentive to honor trust, for instance, in a repeated setting [23, 36]. In a one-shot setting, such a trustee might abuse trust due to the different incentives. Thus, negative information regarding the trustee’s trustworthiness is more salient than positive information. Bolton and colleagues find that trustors in a trust game put more weight on negative than on positive feedback, i.e. trustors more likely withhold trust if the trustee has abused trust beforehand in comparison to trusting a trustee who has honored trust before [31]. We therefore predict negative information has a stronger effect on the trustor not placing trust than positive information has on the trustor placing trust, both compared to the baseline condition without third-party information (hypothesis 1).

### Reliability: personal experiences versus gossip

Information can vary in its reliability and consequently how valuable it is to the trustor. The third party can have information regarding the trustee based on own experiences or based on gossip [37]. According to Granovetter, personal experience is more important to the trustor than gossip. Thus, ‘better than the statement that someone is known to be reliable is information from a third party that he has dealt with that individual and found him so’ [33]. This dimension is usually absent when homepages have official reputation systems, where the buyer must actually have performed a transaction with the seller to be able to give a rating. Third-party information in an informal setting, however, should even strengthen the differences in the value of gossip versus experiences to the trustor. We therefore expect third-party information based on personal experiences to have a bigger impact on placing trust than gossip, compared to the baseline situation with no third-party information (hypothesis 2).

### Source: friend versus stranger

The relation between the trustor and the third party is a continuum reaching from a total stranger to a close friend or family member. The trustor can better judge the importance of the information if she knows the third party very well [38]. If the third party is a stranger, the trustor cannot exclude the possibility that the stranger has a personal interest in misleading the trustor. For instance, the third party might be a close acquaintance of the trustee and have certain incentives to mislead the trustor in order to maintain the relationship with the trustee. Or the third party might be a competitor of the trustee and try to affect the trustee’s reputation by conveying negative information. The trustor cannot know whether this is true if the third party is a stranger to the trustor. A friend, on the other hand, probably has a shared past and a common future with the trustor and therefore has no incentives to provide intentionally wrong information to the trustor. Consequently, the closer the trustor and the informant are, the more likely the given information matters to the trustor and influences her decision whether or not to place trust [32, 39]. Therefore, we expect that information coming from a friend has a stronger effect on placing trust than information coming from a stranger, compared to the situation with no third-party information (hypothesis 3).

### The relative effects of source, reliability, and valence

Our factorial design allows us to study the relative effects of third-party information under different combinations of the source, reliability, and valence of the information. In addition, given either positive or negative information, we can identify any interactions between the source and reliability of third-party information in terms of placing or withholding trust. Our predictions follow partially from the three hypotheses stated earlier.

Let us first focus on the relative effects of positive information from a friend versus a stranger and positive information based on experience versus gossip. We hypothesized that positive experience would be more important to the trustor and consequently have a bigger impact on placing trust than positive gossip. Moreover, positive information from a friend is more salient than positive information from a stranger. When both source and reliability work in the same direction, predictions are clear. We thus expect the positive experience of a friend to have the biggest impact on placing trust and positive gossip from a stranger to have the lowest impact. When source and reliability have countervailing relative effects, however, we cannot predict from our main hypotheses whether positive gossip from a friend or the positive experience of a stranger will be more valuable to the trustor. Nonetheless, our factorial design provides us with the means to empirically measure the relative effects of these two latter combinations.

**Fig 1:**
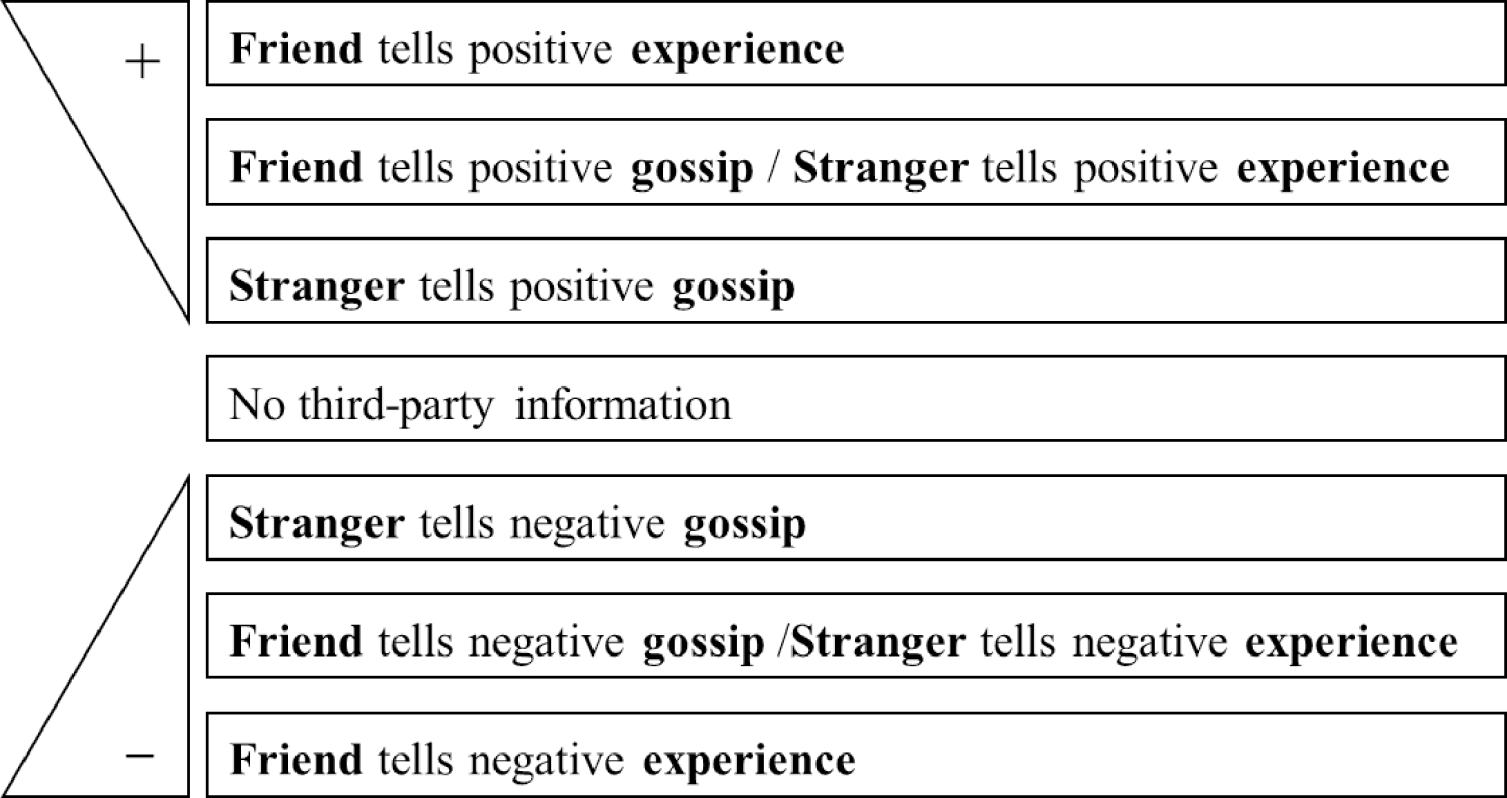
Predicted effects for the interaction of valence, source, and reliability in comparison to the baseline with no third-party information. Fig 1 shows the predicted relative effects on trust for third-party information under different combinations of source and reliability. The triangles on the left side indicate increasing effect sizes in terms of placing trust (+) and withholding trust (-). Each line represents third-party information with a specific set of characteristics in terms of the source and reliability of the information. When both source and reliability operate in the same direction, effects should be relatively large in magnitude (e.g. friend tells experience) or relatively small in magnitude (e.g. stranger tells gossip). When source and reliability work in opposite directions (e.g. friend tells gossip or a stranger conveys an experience), relative effects cannot be predicted a priori. The baseline involves no third-party information.

Now we focus on negative information. Again, source and reliability should both come into play. Again, when source and reliability work in the same direction, predictions are straightforward. We expect the negative experience of a friend to have the biggest impact on not placing trust, and negative gossip told by a stranger to have the smallest impact. We cannot, however, predict whether a friend conveying negative gossip or a stranger sharing a negative experience will affect the decision of the trustor more because source and reliability work in opposite directions. Nonetheless, our factorial design allows us to empirically identify the relative effects of third-party information in these two cases. Fig 1 summarizes the predicted relative magnitude of effects associated with different combinations of source and reliability for both positive and for negative information. The comparison is the baseline vignette with no third-party information.

## Methods

It is basically impossible to isolate the valence, source, and reliability of third-party information from each other outside the laboratory. We therefore study the impact of these three factors on trust with a 2x2x2 within-subjects factorial design that resembles an anonymous internet transaction. Our factorial design uses hypothetical descriptions of situations, often referred to as vignettes, that allow the researcher to vary the factors independently and thus measure their impacts on participant decisions related to the hypothetical situations [40, 41, 42]. Our factorial design has three factors, and each factor has two categories. Specifically, the valence of third-party information can be positive or negative, the source of the information can be a stranger to or a friend of the trustor, and the reliability of the information can be summarized by saying the information is based on gossip or personal experience. Varying these three factors in all combinations leads to eight vignettes. Additionally, we used a baseline vignette with no third-party information as a control. Responding to the control vignette thus required a participant to decide whether to trust given no information about the trustworthiness of the person described in the vignette.

All participants responded to all nine vignettes, which means that each participant responded to the control and to vignettes representing every conceivable combination of valence, source, and reliability. Importantly, we randomly and independently determined the order in which we presented the nine vignettes for each participant. This full factorial design allows us on the one hand to systematically examine the main effects of the three factors on trust. On the other hand, it also allows us to examine the interactions between the three factors in a highly controlled setting. Deciding whether or not to trust someone in a one-shot online transaction involves no clear norm favoring one decision or the other. Thus, our vignettes should not yield socially biased decisions, and therefore demand effects or other biases should be minimal [43].

### Description of the vignettes

The vignette study started with a general description of the situation to the participant. We chose a scenario that resembles a trust situation that is well known to students, namely an online transaction to buy a concert ticket. The participant is asked to imagine looking for two tickets for her favorite concert on the internet because she did not receive tickets via the official ticketing service. An unknown person referred to as A_Schmidt offers to sell the tickets to the participant. The name is chosen to be gender neutral. Moreover, it is a very common surname in German-speaking countries. The vignettes emphasize that the seller is a total stranger to the participant, i.e. to the buyer, and that the participant first has to send the money to the seller before receiving the tickets in return. Thus, the situation resembles a classical trust problem in which participants choose under uncertainty, i.e. not knowing if the trustee is trustworthy or not.

The independent variables of the vignettes included variation regarding the source, the valence, and the reliability of the information about the seller’s trustworthiness. Another person entering the chat introduces third-party information. The third party (i) is either an anonymous guest or a friend of the buyer, (ii) provides information regarding A_Schmidt based on either own experience or gossip, and (iii) provides information either based on a positive or a negative exchange with A_Schmidt in the past. The factorial design consists of three variables with two categories each. Thus, we have eight different vignettes. As a control condition, we add a vignette that does not include third-party information regarding the seller. We therefore have a total of nine vignettes, and each participant responds to the nine vignettes in a random order (see Table 1).

**Table 1:** Overview of the complete 2x2x2 vignette set and the baseline vignette with no third-party information.

Factor combinations	Vignette verbalization
Friend tells positive experience	A friend of the buyer appears in the chat room and says, ‘Hi, I already bought some tickets from A_Schmidt, and the tickets were really sent immediately.’
Friend tells positive gossip	A friend of the buyer appears in the chat room and says, ‘Somebody told me that A_Schmidt will really send the tickets immediately.’
Stranger tells positive experience	A stranger to the buyer appears in the chat room and says, ‘Hi, I already bought some tickets from A_Schmidt, and the tickets were really sent immediately.’
Stranger tells positive gossip	A stranger to the buyer appears in the chat room and says, ‘Somebody told me that A_Schmidt will really send the tickets immediately.’
No third-party information	No third-party appears in the chat room.
Stranger tells negative gossip	A stranger to the buyer appears in the chat room and says, ‘Somebody told me, that A_Schmidt will not send the tickets and will keep the money.’
Stranger tells negative experience	A stranger to the buyer appears in the chat room and says, ‘Hi, I already bought some tickets from A_Schmidt. The promised tickets weren’t sent and the money was kept!’
Friend tells negative gossip	A friend of the buyer appears in the chat room and says, ‘Somebody told me that A_Schmidt will not send the tickets and will keep the money.’
Friend tells negative experience	A friend of the buyer appears in the chat room and says, ‘Hi, I already bought some tickets from A_Schmidt. The promised ticket weren’t sent and the money was kept!’

**Fig 2:**
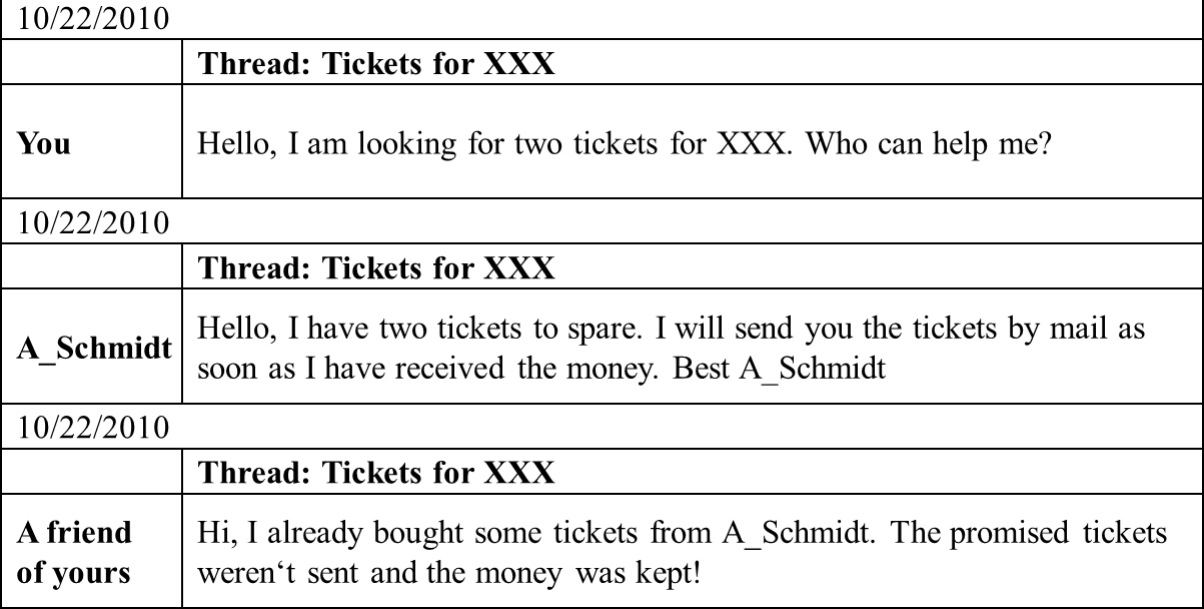
Example vignette of a friend of the buyer (participant) sharing a negative experience with the seller.

In order to make the vignette very realistic and help participants to imagine buying a concert ticket online, we presented the vignettes as an actual exchange in a hypothetical chat room (see for an example Fig 2). This illustration comes close to a real chat room, with the date, the username of the seller, and the title of a thread under which people posts requests. We always set the date a few days after participants were invited to join the survey. The thread is ‘tickets for XXX’. We left the name of the band up to the participant’s imagination, allowing participants to take their own taste in music into account.

### Experimental procedure

We collected data in Switzerland and Germany. Participants from the subject pool of the Department of Economics at the University of Zurich and the Sociology Department of the University of Munich were contacted using the online recruitment tool Orsee [44]. We randomly invited students from each of the subject pools until we had a sufficiently high number of subjects for the study at each location. We excluded students studying psychology, sociology, and economics because in general these students should be familiar with research on trust problems, which could have generated demand effects.

Participants received an invitation to participate in a survey via e-mail. The e-mail explained to them that the study would be online and that the data would be treated anonymously and used for scientific purposes only. All participants knew that they would receive 10CHF/5EURO for the completed questionnaire. Participants understood that the study should not take longer than 20 minutes and that it would be best to fill out the survey without pausing. To reward the participants, we collected addresses at the end of the survey and sent the money via mail. All of this was explained in great detail in the invitation e-mail.

If someone wanted to participate, she would click on a link in the invitation e-mail to sign up for the study. She would then receive another e-mail providing a link for the study itself. Only after clicking on this link could a subject proceed to the study proper. Thus, all subjects provided consent at two separate stages. Moreover, as an online study the subjects completed at home on their personal computers, participation was transparently voluntary. Subjects were obviously free to decide whether and when to exit the study simply by closing the program. Because the option to exit was always present and clearly trivial to implement, and because participation required subjects in effect to repeatedly declare the desire to participate, either by requesting an invitation or by proceeding to the study itself, we treat voluntary participation here as equivalent to written consent. The Human Subjects Committee of the Faculty of Economics, Business Administration and Information Technology at the University of Zurich approved the study.

### Measures

Trust in experimental studies is commonly operationalized as a binary response variable [9, 12, 24, 26, 28] or a continuous response variable [45]. We chose as an individual measure of trust a binary response variable that we present to the subjects directly after each vignette. Given the frame of our vignette study as buying a ticket online, the question of interest is whether or not to enter into a social or economic transaction. Conditional on other variables like the price of the ticket being fixed, this best resembles an intrinsically binary choice. The answers to the question, “What is your decision?” are “Yes, I will send the money and expect to receive the tickets in return,” (value 1) or “No, I won’t send the money” (value 0). To best capture the effects on the binary dependent variable, we estimate logit models and report the average marginal effects (AMEs) for comparison and interpretation [46]. To test the different hypotheses, we created dummies for each of the eight vignettes that include third-party information or in some cases dummies for a combination of vignettes that share relevant characteristics.

Hypothesis (1) predicts a stronger effect of negative information on withholding trust compared to the effect of positive information on placing trust as deviations from the baseline with no information. To test this hypothesis we generate a dummy variable for positive information, referring to a vignette containing positive information independent of the source and the reliability of the third-party information. In parallel, we create a dummy variable for negative information, referring to a vignette containing negative information, disregarding the source and the reliability of the information. The baseline category is always the vignette without any information. To test hypothesis (2) we create two dummy variables. One for the vignettes in which the trustor receives information based on an experience of the third-party, and one for the vignettes in which the third-party only shares gossip. The experience and the gossip dummy contain both positive and negative information. To prevent negative and positive information cancelling each other out, we separate these dummies with regard to positive and negative information. To test hypothesis (3) the trust decision is regressed on dummies such that the third party is either a friend or a stranger to the trustor. However, friends and strangers tell positive and negative information. Therefore, we again separate the dummy variables for positive and negative information.

After subjects finished the vignettes, they filled out a questionnaire. The questionnaire contained several questions in order to elicit control variables. The control variables can be categorized as questions on social value orientation, risk taking, generalized trust, and social capital. Finally, we collected information on age, gender, whether the subject had ever before bought concert tickets in a chat room, whether the subject is member of any online community, and whether the subject participated in Switzerland or Germany. The control variables are described in great detail in S1 Appendix.

## Results

The results section is divided into three parts. First, we present the descriptive and bivariate results. Second, we depict the main results of the multivariate logit models. Third, we check our results with two robustness tests.

### Descriptive results

The sample consists of 69 females and 47 males. The distribution of times to complete the task revealed only three participants who were clearly outliers, and these participants probably paused in the middle of the task. With these three participants, the mean completion time was 32 minutes with a standard deviation of 13 minutes. Without these three participants, the mean completion time was 15 minutes, the standard deviation was six minutes, and the maximum time was 47 minutes. Thus, aside from three outliers, we suspect that the vast majority of participants completed the task without significant pauses. Additional information regarding descriptive statistics for all control variables is presented in S1 Appendix of the supporting materials.

**Fig 3:**
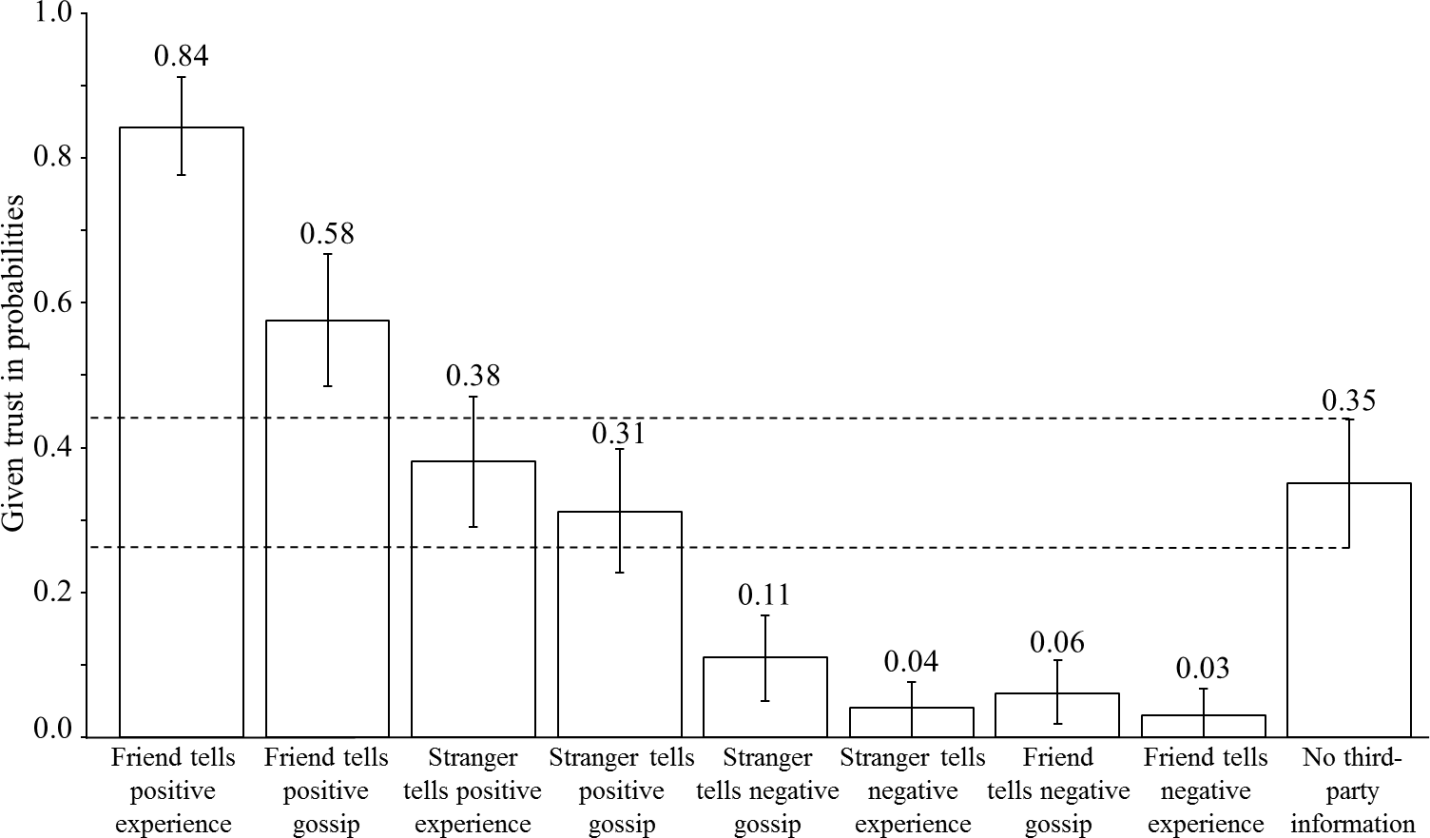
Descriptive Results. Reference category is the vignette ‘No third-party information’.

Over all vignettes, participants trust in 30% of cases. Fig 3 presents the percentage of trust for the different vignettes. If a friend provides positive information, 84% of the subjects are willing to place trust. This reduces to 58% if the friend shares positive gossip. Comparing the 95%-confidence intervals (95%-CIs) we see that both conditions are significantly different from the situation with no information (35%). This changes if positive information comes from a stranger. Then participants place trust in 38% of all cases if the stranger reports her own experiences, and 31% if the stranger shares gossip. With respect to the 95%-CI, positive information from a stranger is not significantly more important than having no information, independent of whether the stranger provides positive experience or positive gossip. If information is negative, 3% of the participants trust if a friend shares experiences, 6% if a friend shares gossip, 4% if a stranger shares experiences, and 11% if a stranger shares gossip. Thus, any information on the untrustworthiness of the trustee makes the participants less likely to place trust, independent of the source or the reliability of the information. All four vignettes in the negative domain lower the trust rate significantly compared to the situation with no information.

### Multivariate Analysis

In further multivariate analyses we ran logit models including all control variables (listed in S1 Appendix). In all models we clustered the robust standard errors (in parentheses) on subject and reported the average marginal effects (AMEs). Three of the control variables (S1 Appendix) had a significant influence on trust at the 5%-level. Namely, subjects that had bought an online ticket before trusted more than those who had not. Germans trusted less than Swiss, and prosocial types, as measured by social value orientation, were more trusting than competitive types.

The first hypothesis to test is the difference between positive and negative information. We expected that negative information has a stronger effect on not placing trust than positive information has on placing trust. We control for negative and positive information with the reference category no information in a simple logit model. For the comparison of the coefficients, we conducted a Wald test with the hypothesis in both models: β_positive_ = (−1) * β_negative_. Table 2 shows the coefficient and the χ^2^-value of the Wald test.

**Table 2:** Valence of information.

Dependent variable: Trust	Logit model (AMEs)
Positive information	0.12***
	(0.03)
Negative information	-0.34***
	(0.035)
Control variables	yes
Observations	1044
Subjects	116
Pseudo-R^2^	0.27
χ^2^-Wald	15.27***

Base category is ‘No third-party information’.

*** p < 0.001

Compared with the no information situation, positive and negative information have significant effects in the expected directions. The average marginal effects of the logit model imply an average reduction of trust of around 34% points if the trustor receives negative information. Positive information on the contrary increases the probability to trust around 12% points. The Wald test is highly significant, showing that positive information has a weaker effect on placing trust than negative information has on not placing trust (χ^2^ = 15.27; p < 0.001).

The second hypothesis on the reliability of information predicts that experience has a stronger effect than gossip on the trustor’s decision to trust, in comparison to the situation with no information. The hypothesis is tested separately for positive and negative information. Again we conduct a simple logit model, and we test the differences between the coefficients with a Wald test. Table 3 presents the results of the regression.

**Table 3:** Reliability of information.

Dependent variable: Trust	Logit model (AMEs)
Positive experience	0.17***
	(0.03)
Positive gossip	0.06*
	(0.03)
Negative experience	-0.40***
	(0.05)
Negative gossip	-0.28***
	(0.04)
Control variables	yes
Observations	1044
Subjects	116
Pseudo-R^2^	0.28
χ^2^-Wald_*positive*_	27.53***
χ^2^-Wald_*negative*_	7.45**

Base category is ‘No third-party information’.

*** p < 0.001

** p < 0.01

* p < 0.05

As expected, positive experience leads to about 17% points more trust, and positive gossip increases the trust rate about 6% points. Negative experience and negative gossip reduce trust by 40% points and 28% points respectively. The differences between the experience and the gossip coefficients are significant for positive and for negative information. In the negative domain the difference is not as clear as in the positive domain. The χ^2^-values for positive information (χ^2^ = 27.53, p < 0.001) are larger than those for negative information (χ^2^ = 7.45, p = 0.006).

Table 4 shows the results for testing the third hypothesis on the source of information, which expects information from a friend to have a larger effect on the trustor’s behavior than information from a stranger. Our analysis was based on the same strategy as the models above. If the trustor receives positive information from a friend, her willingness to trust rises about 23% points compared to the baseline without third-party information. We find no effect on the trustor’s willingness to trust, if positive information comes from a stranger comparing to the baseline condition. Interestingly, negative information always reduces the trust rate significantly and independently of the source of the information. Negative information from a friend reduces the willingness to trust by 35% points, and negative information from a stranger by 27% points. The source of the information only matters to the trustor if the information is positive (χ^2^ = 65.59; p < 0.001), it does not matter for negative information (χ^2^ = 3.03, p = 0.082).

**Table 4:** Source of information.

Dependent variable: Trust	Logit model (AMEs)
Positive information from a friend	0.23***
	(0.03)
Positive information from a stranger	0.01
	(0.03)
Negative information from a friend	-0.35***
	(0.04)
Negative information from a stranger	-0.27***
	(0.04)
Control variables	yes
Observations	1044
Subjects	116
Pseudo-R^2^	0.37
χ^2^-Wald_*positive*_	65.59***
χ^2^-Wald_*negative*_	3.03

Base category is ‘No third-party information’.

*** p < 0.001

**Fig 4:**
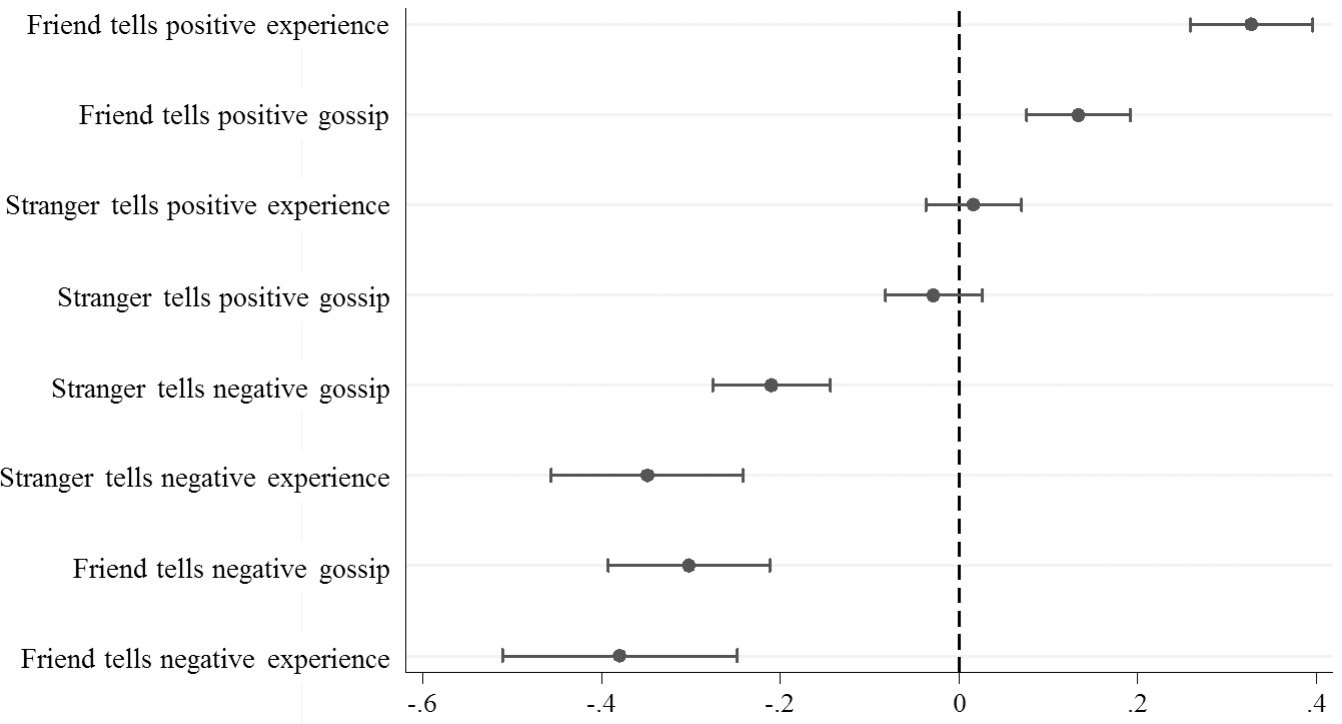
The average marginal effects on the trust decision. In Fig 4 the vertical axis shows the predicted relative effects on trust of the valence (negative versus positive), source (stranger versus friend), and reliability (gossip versus experience) of third-party information. All eight possible combinations are shown, and they are ranked according to the predictions in Fig 1. As discussed in the caption to Fig 1, the cases in which source and reliability operate in opposite directions have no clear ranking, and here we have simply chosen to posit a stronger effect for a friend with gossip compared to a stranger with experience. The horizontal axis shows the average marginal effects (AMEs) with confidence intervals as estimated from our data. The vertical line at zero represents the baseline category in which no third-party information was available.

Fig 4 shows the relative magnitudes (AMEs) of the interactions of the factors on placing and withholding trust based on a simple logit model. One particularly interesting result shows the predicted relative effects, based on the source and reliability of the third-party information, held for positive information but not for negative information. In particular, for negative information we do not find effects that vary based on source and reliability. Instead, regardless of source and reliability, the data show that even the slightest hint of facing an untrustworthy trustee leads to significantly less trusting than having no information.

All comparisons of the coefficients are tested with Wald tests. The χ^2^-values and the significance level are reported in parentheses. We focus first on the relative effects for positive information. A friend’s positive experience has the biggest coefficient and is, as expected, significantly larger than friend‘s gossip and stranger’s experience (χ^2^ = 29.82, p < 0.001, and χ^2^ = 55.36, p < 0.001). The relative effects of a friend’s positive gossip versus a stranger’s positive experience cannot be predicted a priori. However, the Wald test shows that the positive gossip of a friend has a stronger effect on placing trust than the positive experience of a stranger (χ^2^ = 12.37, p < 0.001). The factorial design results suggest that the source of positive information is of more salience to the trustor than its reliability. Further, we expected that a friend’s positive gossip and a stranger’s positive experience would both have a larger effect on a trustor’s decision to place trust than a stranger’s positive gossip. This is true for a friend’s positive gossip (χ^2^ = 30.70, < 0.001), but it is only true for stranger’s positive gossip at the 10% significance level (χ^2^ = 3.62, p = 0.057). Additionally, we expected all four positive vignettes to be more important to the trustor than having ‘no information’. ‘No information’ is the base category and corresponds to the zero line in Fig 4. A stranger’s positive experience and a stranger’s positive gossip both overlap this line with their 95%-CI. Thus, positive information by a stranger, independent of the reliability, is equivalent to having no information.

The relative effects regarding negative information are quite different. As expected, a stranger’s negative gossip is more valuable for the trustor for not placing trust than having ‘no information’ at all. The 95%-CI for stranger’s negative gossip does not overlap the zero line. Furthermore, a stranger’s negative experience and friend’s negative gossip matter more than a stranger’s negative gossip (χ^2^ = 8.17, p = 0.004 and χ^2^ = 6.00, p = 0.014). The relative effects of a friend’s negative gossip versus a stranger’s negative experience cannot be predicted a priori. However, the Wald test shows that they do not differ in their significance to the trustor (χ^2^ = 0.66, p = 0.415). A friend’s negative experience does not, as expected, have the biggest effect on withholding trust. A stranger’s negative experience and a friend’s negative gossip are not significantly different from a friend’s negative experience (χ^2^ = 0.14, p = 0.707 and χ^2^ = 1.26, p = 0.262). Thus, any kind of negative information leads to significantly less trust by the trustor than having no information at all. However, the different kinds of negative information do not differ in the value they have for the trustor.

### Robustness checks

The order in which vignettes were presented was independently and randomly determined for each participant. In principle, this procedure ensures that the differences in content between the vignettes, which are our primary interest, should not be systematically related to the order in which the vignettes were presented. By extension, this excludes the possibility that the observed effects of the vignettes on trust were actually due to the order of presentation or due to associated learning effects. Nevertheless, to check for robustness, we re-estimated our logit models with fixed effects at the participant level. This controls for all invariant participant-specific variables. Because each participant was actually presented with the nine vignettes in a unique order, our fixed-effects models thus control, in addition to all other participant-specific characteristics, for the order of presentation and any associated learning effects. Fixed-effects logit models lead to exactly the same conclusions as the simple logit models with clustered standard errors (S1 Appendix). This finding, when coupled with the fact that we randomized the order in which vignettes were presented for each participant, ensures that our findings cannot be attributed in any way to the order of presentation.

Although we can safely exclude order effects, one could nonetheless argue that presenting each participant with all nine vignettes allows participants to eventually infer the research question as they become aware of the full factorial design. If so, our results could conceivably be due to experimental demand effects, which would essentially mean that participants produced responses consistent with what they thought the researchers wanted. To see if this is so, we re-estimated all regression models using only the first four vignettes presented to each participant. Because each participant responded to the vignettes in a random order, we can limit attention to the first four vignettes presented but still estimate effects for all nine vignettes (for information on why we chose the first four vignettes, see supporting information). In effect, all nine vignettes are represented in the data even though we only consider the first four presented to each participant. In addition, because we limit attention to these first four, each participant did not have enough information to know the full design, and hence demand effects are extremely unlikely. Even though the 95%-CIs are naturally larger due to the reduced amount of data, the main findings regarding the direction of the coefficients stay more or less the same. We only observe a slightly reduced significance of the effects (S1 Appendix). This finding shows that our findings are unlikely due to experimental demand effects.

## Discussion

The present research extends previous work on the influence of third-party information regarding beliefs about whether a trustee is trustworthy. By varying the importance of third-party information in terms of the source, the reliability, and the valence to the trustor, the results of the study deepen our understanding of how multidimensional information affects trustors’ decision to trust in anonymous one-shot transactions.

Our factorial design study sheds new light on the question of what kind of third-party information matters most to the trustors [35]. We found a specific ranking of effects associated with positive information such that a friend‘s experience has the strongest effect on placing trust followed by a friend‘s gossip. Interestingly, positive information coming from a stranger rather than a friend, independent of the reliability of the information, is not significantly different from having no information. Regarding negative information, even the slightest hint of an untrustworthy trustee leads to a significant reduction in trust, independent of whether the negative information comes from a friend or a stranger, and independent of whether the information is based on gossip or experiences.

Our factorial design is based on third-party information provided by a single friend or stranger. Official reputation systems provide stranger information based on several hundred or thousands of feedbacks. The effect of negative information then most likely becomes even stronger. Thus, our results show how important it is for huge e-commerce platforms to ensure a fair feedback mechanism based on sellers’ and buyers’ experiences. Most big e-commerce platforms allow removal of feedback under extenuating circumstances. Ebay, for instance, claims on their homepage that ‘[…] we also don’t allow buyers to use feedback in a way that would impact seller performance ratings when sellers have objectively met satisfactory performance levels’ (www.eBay.com). This also explains the tremendous efforts hotels put in replies to negative feedback on feedback homepages like travelocity (www.travelocity.com) or tripadvisor (www.tripadvisor.com). Travellers can rank hotels in terms of service, rooms, and location. Quite often, after receiving a negative ranking, the hotel management replies to the feedback. In more severe cases hotels and other companies have even been blackmailed with bad reputations. Hotel guests have threatened hotels with bad reviews to demand free upgrades or refunds. Tripadvisor recently started a system for hotels faced with blackmails from guests.

This study allows us to compare the significance of third-party information to the trustor based on whether the information comes from a friend or a stranger. Earlier studies either focused on reputation effects based on third-party information from friends by studying social networks [47, 48] or on reputation effects in e-commerce based on feedbacks provided by hundreds of semi-anonymous buyers [1, 15, 19]. We find that information coming from a friend has a larger effect than information coming from a stranger. However, this only holds for positive information and not for negative information. Thus, regarding positive information, people surely trust information from a friend, regardless of the reliability of the information. In the negative domain people do not distinguish between a friend or a stranger as the source of third-party information. Strangers might have an incentive to make a trustor buy from a bad seller because, for instance, the stranger and the seller are acquaintances. This reasoning, however, does not apply with negative information. This might be the reason why we do not find a difference between third-party information provided by a friend or a stranger in the negative domain.

The third party reporting experience with the seller has a stronger effect on the trustor’s decision to place trust than third-party information based on mere gossip. This effect is stronger in the positive than in the negative domain. This shows how important it is that official reputation systems allow people to give feedback only after they have had an actual transaction with the seller. This also undermines fraud. It is harder for the seller to engineer his own positive reputation. We believe that understanding how different kinds of third-party information matter to trustors also helps us to understand the best way to design a formal reputation mechanism on huge internet platforms.

Although our study addresses a topic with no clear norm about whether to trust, and hence social desirability biases should not play a major role, it would nonetheless be extremely useful to study actual internet transactions to test the robustness of our results. Vignette studies with hypothetical incentives always run some risk that observed choices will not carry over to settings with real material incentives [40]. Therefore, it would be very important to run additional studies measuring behavior in settings with material incentives. One way to do this would be to study actual internet transactions with a content analysis of associated messaging. In particular, messages regarding transactions conducted on homepages without a formal system for reputation management could further improve our understanding of what kind of third-party information matters to potential trustors.

Regarding negative information, it would be very useful to study whether differences in the source and the reliability lead to differences in the willingness to pay on the buyer’s side. Empirical research has shown that a seller with a negative reputation affects the buyer’s willingness to pay [17]; sellers with a bad reputation usually sell products at a lower price than sellers who have a good reputation. It would be very interesting to see whether this applies to negative information coming from a friend as well as from a stranger, and independent of whether the information is based on experience or gossip.

Online studies like ours have certain advantages and disadvantages in comparison to laboratory studies [49]. Online studies lack the control of the lab regarding who actually fills out the survey and when. In our study, we did not know whether participants filled out the surveys on their own or whether they were distracted, for instance, because they were watching TV or sending text messages. Because of these issues, an interesting complementary approach our study would be run a trust game experiment in the lab with third-party information entering the trust games in real time.

In addition, it has been shown that children and adults vary in terms of how reliable they consider information coming from the internet [50]. We used a standard student population in our vignette study with an average age of 23. Because our focus was on unregulated third-party information online, it would be an important extension to see how age affects the way people respond to third-party information received via online contacts, which are of course increasingly prevalent in modern social exchanges.

Generally, we hope to call attention to the need to further research third-party information in online transactions that are not covered by the reputation systems of huge internet platforms. We believe this helps to understand how multidimensional third-party information in anonymous online transactions affects trustors’ trust in the absence of official reputation systems.

## Supporting Information

S1 Appendix Description of control variables and robustness checks. (PDF)

S1 Data Raw data. (XLS)
