# Supplementary material for: The Impact of Third-Party Information on Trust: Valence, Source, and Reliability
Source: PLoS One. 2016 Feb 16;11(2):e0149542. doi: 10.1371/journal.pone.0149542 (PMC4755612; doi:10.1371/journal.pone.0149542)
Supplement: S1 Appendix — (PDF) [file pone.0149542.s001.pdf]

S1 Appendix

Control variables

We shortly describe how we measured the control variables after participants finished the vignettes. First, we measured subjects' risk aversion [51] and social value orientation [52]. In order to be able to control for participants' general willingness to trust, subjects responded to a trust scale by Yamagishi and Yamagishi [53] based on three different questions ('Generally, you trust other people', 'These days, you cannot rely on anybody', ranging from 1 (I do not agree at all) to 4 (I totally agree); and 'To what kind of degree do you trust strangers?', from 0% to 100%). Several studies show that social capital generates trust [54, 55]. The underlying argument is that people learn to trust through social interactions with common goals such as in neighborhoods or workplaces. Following Li and colleagues, we controlled for three different types of social capital, namely neighborhood attachment, social network, and civic participation [56]. We measured neighborhood attachment with the number of neighbors the subject knows personally, social networks with a dummy variable for having a partner and two indices that account for the family climate and the embeddedness of the subject in friendship networks. To measure family climate we used the following items: 'In my family everybody supports everyone as good as she/he can', 'We often fight, when we see each other', and 'In my family I often feel restricted', ranging from 1 (I do not agree at all) to 4 (I totally agree). To account for friendships we first measured the 'number of persons, you would call in a crisis', second, the 'number of persons, who would lend you more than 100 CHF/Euro without hesitation', and third, the 'number of persons, you would call really close friends'. Civic participation was measured through counting memberships in the following civic organizations: sports clubs, political groups, music groups or bands, community service organizations, religious groups, other social groups [57]. In order to measure the general trust

of participants in official institutions we constructed an index based on the answers to the questions 'How much do you trust the political system, the system of justice, commercial enterprises, Civil Service, the media, the clergy, the educational system, and the social market economy', on a scale from 1 (do not trust at all) to 5 (trust a lot) [57]. Finally, we collected information on age, gender, whether the subject had ever been in the situation of buying concert tickets in a chat room, whether the subject is member of any online community, and whether the subject participated in Switzerland or Germany.

Table A. Descriptive Statistics.

Variables	Description	Mean	SD
**Dependent Variable**			
trust	Dummy variable taking value 1 if the subject decided to trust, 0 otherwise.	0.30	
**SVO and risk**			
prosoc_d	Dummy variable taking value 1 if the subject is a prosocial type, 0 otherwise.	0.36	
compet_d	Dummy variable taking value 1 if the subject is a competitive type, 0 otherwise.	0.09	
indiv_d	Dummy variable taking value 1 if the subject is a individualistic type, 0 otherwise.	0.34	
notyp_d	Dummy variable taking value 1 if the subject is a unclassified type, 0 otherwise.	0.20	
riskaversion	Quasi-metric measurement of risk aversion (from 1 to 11).	6.09	2.12
**Situational variables**			
member_online	Dummy variable taking value 1 if the subject is a member of an online network, 0 otherwise.	0.72	
ever_situation	Dummy variable taking value 1 if the subject has ever been in a similar situation, 0 otherwise.	0.14	
**General trust**			
generaltrust_index	Index out of 3 items to measure general trust attitudes.	2.16	0.41
trust_insitutions_index	Index out of 8 items to measure the subjects trust in different institutions.	2.99	0.57
**Social Capital**			
number_neighbors	Numbers of neighbors personally known.	8.28	9.90
having_partner	Dummy variable taking value 1 if the subject has a partner, 0 otherwise.	0.46	
friends_number	Average number of good friends, who either lend the subject money, who could be called in case of problems or who are considered as good friends.	6.39	4.09
family_index	Index out of 3 items to measure the family climate.	3.11	0.53
memberships	Number of memberships in civic organizations.	1.39	0.99
**Demographics**			
age	Age in years.	23.22	3.15
german	Dummy variable taking value 1 if the subject is from Germany, 0 if the subject is from Switzerland.	0.32	
Observations		1044	
Subjects		116	

Variable names correspond to the names in the data file.

Robustness test with fixed effects at the participant level

Table B. Multivariate Analysis: Valence of information, fixed effects model.

**Dependent variable: Trust**	**Fixed effects logits**
Positive information	1.06***
	(0.27)
Negative information	-3.11***
	(0.39)
Control variables (see Table A)	fixed
Observations	936
Subjects	104
χ^2-Wald	12.63***

Base category is ‘No third-party information’.

∗∗∗ $p < 0.001$, ∗∗ $p < 0.01$, ∗ $p < 0.05$

Table C: Multivariate Analysis: Reliability of information, fixed effects model.

**Dependent variable: Trust**	**Fixed effects logits**
Positive experience	1.54***
	(0.30)
Positive gossip	0.59*
	(0.30)
Negative experience	-3.96***
	(0.57)
Negative gossip	-2.66***
	(0.42)
Control variables (see Table A)	fixed
Observations	936
Subjects	104
χ^2-Wald$_{positive}$	17.30***
χ^2-Wald$_{negative}$	5.85*

Base category is ‘No third-party information’.

$***$ $p < 0.001$, $**$ $p < 0.01$, $*$ $p < 0.05$

Table D. Multivariate Analysis: Source of information, fixed effects model.

**Dependent variable: Trust**	**Fixed effects logits**
Positive information from a friend	2.34***
	(0.34)
Positive information from a stranger	-0.07
	(0.32)
Negative information from a friend	-3.76***
	(0.52)
Negative information from a stranger	-2.98***
	(0.44)
Control variables (see Table A)	fixed
Observations	936
Subjects	104
χ^2-Wald$_{positive}$	67.74***
χ^2-Wald$_{negative}$	2.55

Base category is 'No third-party information'.

∗∗∗ $p < 0.001$, ∗∗ $p < 0.01$, ∗ $p < 0.05$

Fig A. Coefficients on the trust decision (95%-CI), with fixed effects at the participant level.

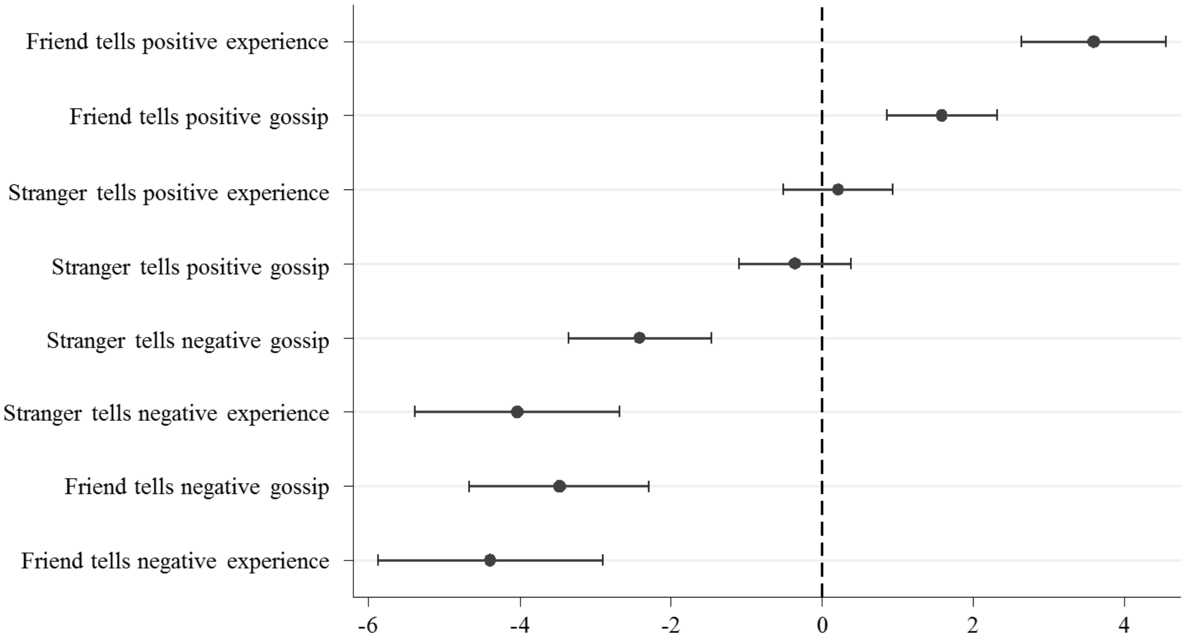

Robustness test with four vignettes

We cannot fit models with only one or two vignettes per subject because of complete or quasi-complete separation. This is a common problem with logistic regressions and small sample sizes, which we necessarily have when we limit attention to only one or two vignettes per subject. In cases of this sort, statistical models cannot be fit to the data because one or more of the independent variables perfectly explains the response variable.

Using three vignettes allows us to fit a model, but the results are not significant, presumably because of limited power. Using four vignettes allows us to fit a model, and the results are significant in the same way as the full analysis in the main paper. In any case, the patterns that characterize trust are always the quite same regardless of whether we focus on one, two, three, four or nine vignettes (Table E, F, G, and Fig B).

Table E. Multivariate Analysis: Valence of information, first four vignettes presented.

Dependent variable: Trust	Logit model (AMEs)
Positive information	0.18***
	(0.05)
Negative information	-0.26***
	(0.060)
Control variables (see Table A)	yes
Observations	464
Subjects	116
Pseudo-R^2	0.27

Base category is ‘No third-party information’.

∗∗∗ $p < 0.001$, ∗∗ $p < 0.01$, ∗ $p < 0.05$

Table F. Multivariate Analysis: Reliability of information, first four vignettes presented.

Dependent variable: Trust	Logit model (AMEs)
Positive experience	0.22***
	(0.05)
Positive gossip	0.01*
	(0.05)
Negative experience	-0.35***
	(0.09)
Negative gossip	-0.20**
	(0.07)
Control variables (see Table A)	yes
Observations	464
Subjects	116
Pseudo-R^2	0.28

Base category is ‘No third-party information’.

*** $p < 0.001$, ** $p < 0.01$, * $p < 0.05$

Table G. Multivariate Analysis: Source of information, first four vignettes presented.

Dependent variable: Trust	Logit model (AMEs)
Positive information from a friend	0.27***
	(0.04)
Positive information from a stranger	0.03
	(0.04)
Negative information from a friend	-0.32***
	(0.08)
Negative information from a stranger	-0.18**
	(0.06)
Control variables (see Table A)	yes
Observations	464
Subjects	116
Pseudo-R^2	0.34

Base category is ‘No third-party information’.

*** $p < 0.001$, ** $p < 0.01$, * $p < 0.05$

Fig B. Average marginal effects on the trust decision (95%-CI), first four vignettes presented. Control variables see Table A.

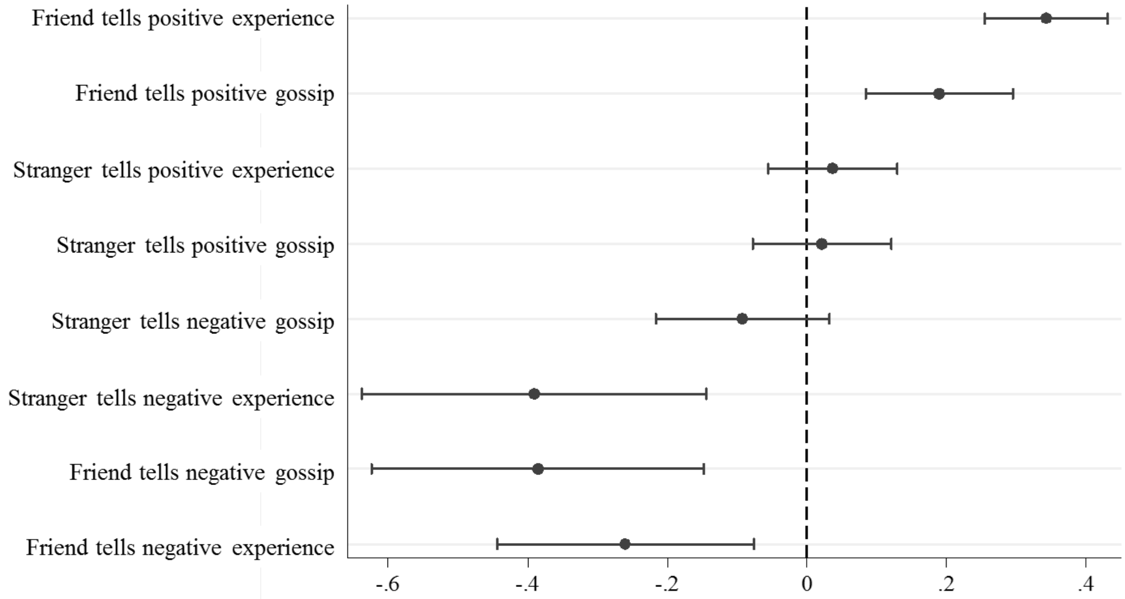

References of appendix

51. Wagner G, Frick J, Schupp J. The German socio-economic panel study (GSOEP) – Evolution, scope and enhancements. Schmollers Jahrbuch. 2007; 127(1): 139-170.
52. Van Lange P. The pursuit of joint outcomes and equality in outcomes: an integrative model of social value orientation. Journal of personality and social psychology. 1999; 77(2): 337-349.
53. Yamagishi T, Yamagishi M. Trust and commitment in the United States and Japan. Motivation and emotion. 1994; 18(2): 129-166.
54. Glaeser EL, Laibson D, Scheinkman JA, Soutter CL. What is social capital? The determinants of trust and trustworthiness. Nber Working Paper 7216; 1999.
55. Putnam RD. Bowling alone: the collapse and revival of American community. New York, NY: Simon and Schuster; 2000.
56. Li Y, Pickles A, Savage M. Social capital and social trust in Britain. European sociological review. 2005; 21(2): 109-123.
57. Paxton P. Is social capital declining in the United States? A multiple indicator assessment. American journal of sociology. 1999; 105(1): 88-127.
